# Accuracy of an internet-based speech-in-noise hearing screening test for high-frequency hearing loss: incorporating automatic conditional rescreening

**DOI:** 10.1007/s00420-018-1332-5

**Published:** 2018-06-29

**Authors:** Marya Sheikh Rashid, Wouter A. Dreschler

**Affiliations:** Department of Clinical and Experimental Audiology, ENT Department, Academic Medical Center (AMC), Meibergdreef 9, 1105 AZ Amsterdam, The Netherlands

**Keywords:** Occupational high-frequency hearing loss, Noise-induced hearing loss, Screening, Speech-in-noise, Internet, Test sensitivity and specificity

## Abstract

**Purpose:**

To validate the accuracy of an internet-based speech-in-noise hearing screening test for high-frequency hearing loss (HFHL) ‘Occupational Earcheck (OEC)’ incorporating an automatic conditional rescreening, in an occupationally noise-exposed population. Secondary objectives were to assess the effects of age on test accuracy measures, and to assess the test accuracy for different degrees of HFHL.

**Methods:**

A study was conducted on cross-sectional data of occupational audiometric examinations, including the index test OEC and reference standard pure-tone air conduction audiometry, of 80 noise-exposed workers. Sensitivity, specificity, and likelihood ratios were calculated for the OEC, after automatic conditional rescreening, for a younger and an older age group, and for two degrees of HFHL (HFHL_25_: PTA3,4,6 ≥ 25 dB HL, and HFHL_35_: PTA3,4,6 ≥ 35 dB HL, both for at least one ear).

**Results:**

Test specificity for HFHL_25_ after a single test was 63%, and improved to 93% after the automatic conditional rescreen. Test sensitivity for HFHL_25_ decreased from 65% to 59%. Test sensitivity and specificity including automatic conditional rescreening for HFHL_35_ was 94% and 90%, respectively. The positive likelihood ratio for HFHL_25_ was 8.4, and for HFHL_35_ 9.4. The negative likelihood ratio for HFHL_35_ was below 0.1.

**Conclusions:**

The OEC is an appropriate screening test, especially for HFHL_35_. Normal-hearing workers who obtained a positive test result for the first test for one or two ears, benefit from having an automatic rescreen, resulting in an improvement of the test specificity, and hence prevent unnecessary referral.

## Introduction

High-frequency hearing loss (HFHL), caused by excessive exposure to noise in the workplace, also known as noise-induced hearing loss (NIHL), is an important public health problem worldwide (May 2000; Sliwinska-Kowalska and Davis 2012). In the Dutch construction industry, it is one of the most commonly reported occupational diseases (van der Molen et al. 2016). Therefore, secondary prevention (i.e., early identification) of HFHL by screening is of great importance, and stimulates to take actions to prevent progression of the hearing loss (Meyer-Bisch 1996).

Over the past few years several internet-based speech-in-noise self-tests have been developed and investigated (Smits et al. 2006; Jansen et al. 2010; Leensen et al. 2011b; Watson et al. 2012; Molander et al. 2013; Paglialonga et al. 2014; Vlaming et al. 2014; Williams-Sanchez et al. 2014). Studies have shown that these tests can be used as a proper screening tool (Smoorenburg 1992; Smits et al. 2004, 2006, 2013; Culling et al. 2005; Jansen et al. 2010; Leensen et al. 2011b). These tests facilitate audiometric hearing evaluation of noise-exposed workers in the workplace: a trained audiometrist, a soundproof room, and specialized, and costly technical equipment are no longer required, as is the case for the more conventional pure-tone air conduction screening audiometry (Stenfelt et al. 2011; Leensen and Dreschler 2013a).

This study focuses on the Occupational Earcheck (OEC), a Dutch internet-based speech-in-noise hearing screening test for occupational HFHL, developed at the Department of Audiology of the Leiden University Medical Center, commissioned by the Netherlands Hearing Health Foundation (Ellis et al. 2006). A phased approach was maintained to evaluate this test for screening purposes in noise-exposed workers. In the first phase, the concept was improved for HFHL, and tested in a well-controlled laboratory setting in a population that was recruited by means of a two-gate design, with normal-hearing cases on the one hand, and known HFHL cases on the other (Sheikh Rashid et al. 2017a). In the second phase, the improved test was evaluated in an unselected group of noise-exposed employees in a quiet office room at the work place (Sheikh Rashid et al. 2017b). The discriminative ability of OEC was calculated on the individual level, which means that the results of both ears were taken into account. Based on the classification of HFHL for at least one ear versus no HFHL for both ears, the sensitivity on the individual level was 90% and the specificity was 77%. A relatively large measurement error was found, possibly due to a learning effect between the single ear measurements within one test. The learning effect may have led to higher estimated SRT values, especially for the first ear measured, and the relatively high number of false-positive HFHL classifications. Though learning was accounted for by training, and a long individual run-up to the actual measurement was incorporated in the test, a learning effect still appeared.

In a screening setting, even a small learning effect may result in an incorrect classification due to the dichotomous test outcome. Normal-hearing listeners who have trouble with understanding the test procedure or who are not yet familiar with the speech material, may incorrectly receive a positive test score. A potential solution to this problem is to provide a second test opportunity for the initial referrals. Listeners may benefit from an automatically offered rescreen, provided for the ear(s) with a poor result, as the final classification (pass or referral) will be based on the last test result.

The objective of this study was to validate the test accuracy of OEC incorporating a new procedure with an automatic conditional rescreening, in a representative study population of noise-exposed workers. Test accuracy measures, including sensitivity, specificity, predictive values, and likelihood ratios were calculated. Secondary objective was to assess the effect of automatic conditional (i.e., sequential) rescreening of the positives on test accuracy measures. Another secondary objective was to establish the test accuracy for different degrees of HFHL, and for different age groups.

## Methods

### Study population

The study population consisted of occupationally noise-exposed employees from two manufacturing companies in the Netherlands who voluntarily performed an occupational audiometric examination provided by their employers, which is according to the Dutch Working Conditions Act. Subjects were 18 years or older and were speakers of the Dutch language. There were no exclusion criteria. The employees were informed by their employer by means of an information letter, and gave approval for sharing their results with researchers of the Amsterdam Medical Center for research purposes. According to the Medical Ethics Committee of the University of Amsterdam official approval of this study was not necessary, as the Medical Research Involving Human Subjects Act does not apply to this study (reference number W17_254 # 17.297).

### Measurement procedure

This prospective cross-sectional study was based on data from occupational audiometric examinations of noise-exposed workers that were performed in 2016. For every employee results of the index test OEC were collected. As a reference, pure-tone air conduction thresholds were collected by means of pure-tone air conduction audiometry. Demographical data on gender and age were collected.

### Occupational Earcheck

The speech material of OEC consisted of a closed set of eight equally intelligible Dutch consonant–vowel consonant (CVC) words with matched vowels, represented by eight response buttons on a visual screen, identified by a picture and a written word. A ninth button labelled ‘not recognized’ was included. The speech material was presented in a stationary low-pass filtered masking noise. Test presentation was monotic; both left and right ear were tested separately. The sequence of the ears was randomly assigned by OEC. The first stimulus was presented at a signal-to-noise ratio (SNR) of 0 dB, and with every correct response the subsequent stimulus level was decreased by 2 dB, while with every incorrect answer it was increased by 2 dB. The noise level remained fixed throughout the test. The noise level could be set by the user to a comfortable loudness by means of a volume scale, resulting in individual test intensities. The actual measurement started at the SNR of the first incorrect response, resulting in an individual starting level. Total test length per ear measurement was shortened to 20 presentations. The speech-reception threshold (SRT) was calculated by averaging the SNRs of the last ten stimuli. The intra-test standard deviation (SD) of the last ten stimuli gave an insight into the variation within a single test measurement. The previously established cut-off value of − 14.9 dB SNR was used for pass/fail (Sheikh Rashid et al. 2017b). To achieve a good (i.e., negative) result for OEC, a subject would need a SRT score of < − 14.9 dB SNR for both ears. A subject would get a poor (i.e., positive) result for OEC if the test result of at least one ear was ≥ − 14.9 dB SNR. More details on the development of OEC are described elsewhere by Sheikh Rashid et al. (2017a, b).

The test was performed on an Apple Ipad with on-ear HQ-HP113LW headphones in a quiet office room at the work setting. OEC self-tests were minimally supervised by testers of the Netherlands Hearing Health Foundation. The testers were not aware of the results of the pure-tone air conduction audiometry. A sequential test design was applied. Listeners with a positive test result on the first test, automatically received a rescreen. The rescreening was conditional: a retest was only provided for the ear(s) with a positive test result, or with an intra-individual SD of > 3 dB. Based on previous research, test results with an intra-individual SD of > 3 dB were considered unreliable (Sheikh Rashid et al. 2017a).

### Pure-tone air conduction audiometry

Pure-tone air conduction audiometry was performed by professional audiometrists in sound-insulated office cabins, with ambient sound levels of 31 and 34 dBA, at both work settings, with the use of the clinical audiometers Madsen Micromate 304 (Otometrics) and Voyager 522, connected to TDH39 headphones. The headphones were provided with sound-attenuating Amplivox audiocups, because it could not be guaranteed that the audiometric test conditions of the office cabins met the international standards for hearing screening (i.e., unmasked air conduction starting at 500 Hz; ISO 8253, part I, 2010). The audiometers were calibrated and were in compliance with the norm EN 60645-1 (ANSI S3.6, Type 2). Pure-tone air-conducted hearing thresholds were collected for both ears for the octave frequencies between 0.25 and 8 kHz (and additionally for 3 and 6 kHz). The audiometrists were not aware of the OEC results of the workers.

### Statistical analyses

Descriptive statistics were performed on demographic information, and pure-tone thresholds. True HFHL on the basis of pure-tone air conduction audiometry was defined as a pure-tone average (PTA) of the frequencies 3, 4, and 6 kHz (PTA_346_) of 25 dB HL or worse for at least one ear (HFHL_25_). A second, higher, degree of HFHL was defined as a PTA_346_ of 35 dB HL or worse for at least one ear (HFHL_35_). When thresholds for certain frequencies were missing, the adjacent thresholds were interpolated. Two-by-two contingency tables were used to compare the performance of OEC with pure-tone air conduction audiometry. Test properties were calculated, including sensitivity and specificity^1^, positive and negative predictive values^2^, and positive and negative likelihood ratios^3^ (sensitivity/1 − specificity, and 1 − sensitivity/specificity), for the single screen versus the conditional rescreen, for two degrees of HFHL, and for separate age groups. To assess the effect of age, the workers were divided into a younger age group (≤ 45 years), and an older age group (> 45 years). Likelihood ratios were calculated to overcome the disadvantage of a single cut-off value, and to apply the results of OEC to the individual (Parikh et al. 2009), making them useful for screening practice. Data were analyzed using IBM SPSS Statistics 24.

## Results

**Fig. 1:**
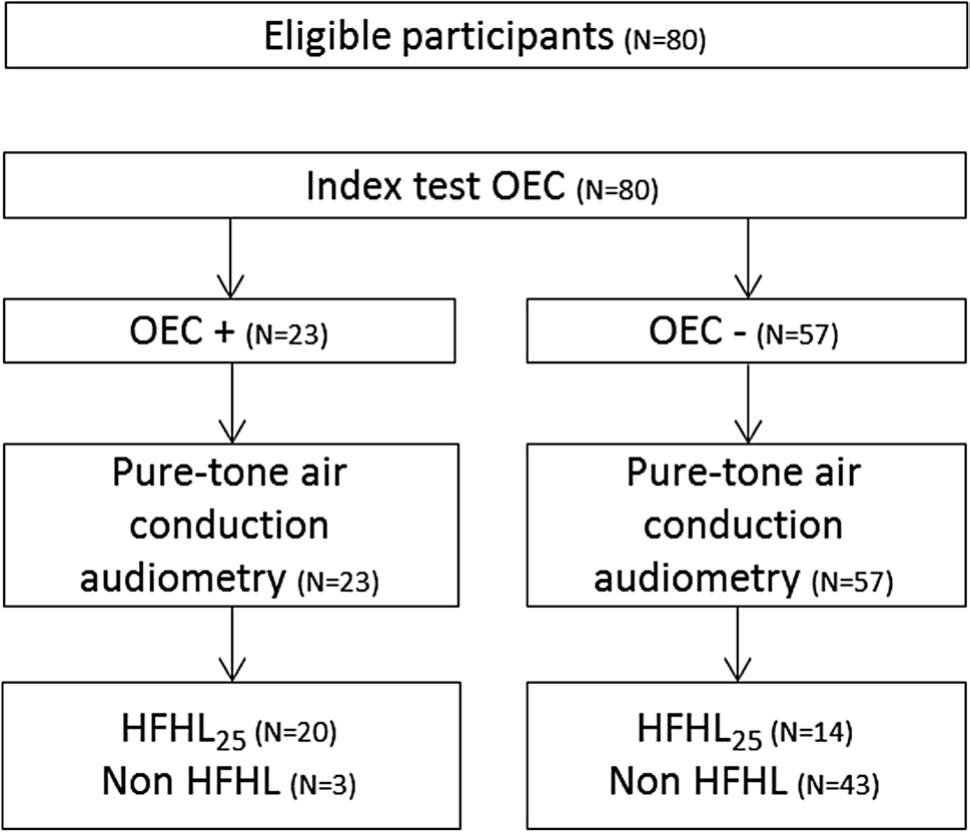
STARD diagram, with classification based on results of automatic rescreen. Index test is OEC. Reference test is pure-tone air conduction audiometry. Target condition is HFHL_25_ (for at least one ear). *N* number of participants, *OEC* Occupational Earcheck, *HFHL* high-frequency hearing loss

**Fig. 2:**
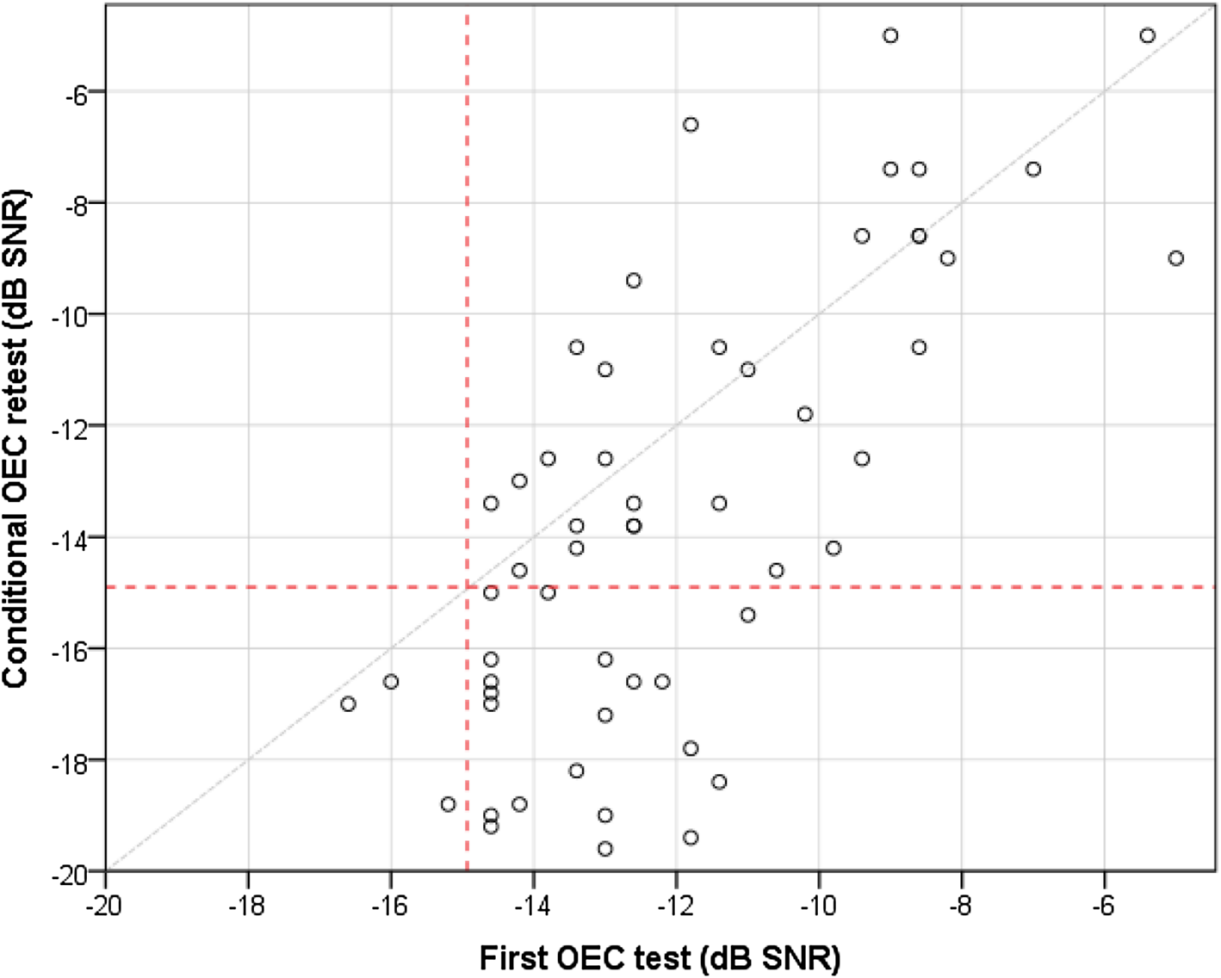
Scatterplot of (first) OEC test and retest (rescreen) results for all retested ears (*N* = 55). The horizontal and vertical interrupted lines depict the cut-off value for pass/fail, set at − 14.9 dB SNR

**Fig. 3:**
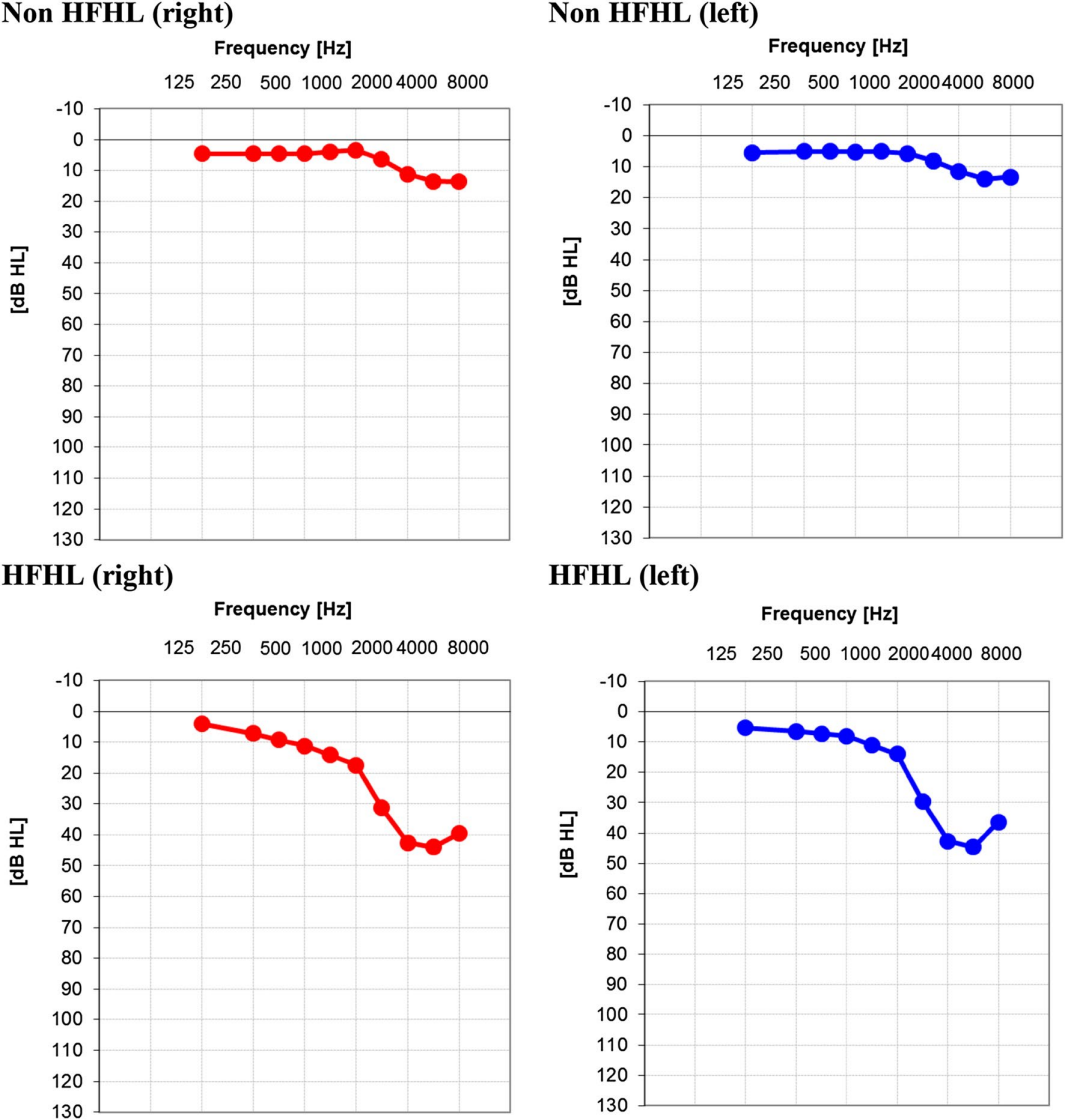
Mean pure-tone air conduction audiometry thresholds for non-high-frequency hearing loss (non HFHL) ears (upper panels) and for ears with high-frequency hearing loss defined as a pure-tone average of the frequencies 3, 4, 6 kHz (PTA_346_) of 25 dB HL or worse (HFHL_25_) (lower panels). The thresholds for left and right ears are presented separately

In total, data of 80 noise-exposed workers were available. All workers performed the index test (OEC) and the reference test (pure-tone air conduction audiometry). A STARD diagram is given in Fig. 1, to report the flow of participants in the study. We could not analyze the effects of gender because the vast majority of the subjects were male [*N* = 78 (97.5%)]. The mean age was 44.0 years (SD = 11.5). About half of the participants underwent a rescreen for at least one ear (*N* = 42 (52.5%)). In total, 55 ears were rescreened, of which 52 ears with a positive test result (8 of these ears also had an intra-individual SD > 3 dB). Three ears with a negative test result were rescreened due to an intra-individual SD > 3 dB. Figure 2 presents a scatterplot of first test and rescreen results for all ears that were retested. The prevalence of HFHL_25_ (for at least one ear) was 42.5% (34 out of 80 workers). Four workers (5%) had a HFHL_25_ at the right ear only, and nine (11.3%) workers had a HFHL_25_ for the left ear only. Twenty-one workers (26.3%) had a HFHL_25_ for both ears. The remaining 46 subjects (57.5%) showed normal results on both ears at the OEC test. Figure 3 presents mean hearing thresholds of both ears, for non HFHL_25_ individuals, and individuals with HFHL_25_ for at least one ear. The prevalence of HFHL_35_ for at least one ear was 22.5% (18 out of 80 workers). The group of ≤ 45 years (*N* = 41) had a mean PTA_346_ of 12.8 dB HL (SD = 13.5) for the right ear and 15.0 dB HL (SD = 15.0) for the left ear. The older age group (*N* = 39) had a mean PTA_346_ of 26.3 dB HL (SD = 16.6) for the right ear and 28.4 dB HL (SD = 15.3) for the left ear. The differences between the younger and the older group in mean PTA_346_ for both the left ear and the right ear were statistically significant (*p* < 0.001).

The mean SRT score based on the single screen was − 15.5 dB SNR (SD = 3.1) for the right ear, and − 15.5 dB SNR (SD = 3.3) for the left ear. The mean intra-individual standard deviation was 2.0 dB for both the left ear and the right ear. The mean SRT score including the conditional rescreen was − 16.2 dB SNR (SD = 3.1) for the right ear and − 16.0 dB SNR (SD = 3.2) for the left ear. The mean intra-individual standard deviation for the right ear was 1.9 dB (SD = 0.6) and for the left ear 2.0 dB (SD = 0.6). The correlation coefficient for PTA_346_ and OEC results including conditional rescreen was *r* = 0.57 for the right ears (*p* < 0.01) and *r* = 0.61 for the left ears (*p* < 0.01).

Table 1 presents the OEC results (positive for at least one ear versus negative for both ears) compared to pure-tone air conduction audiometry results (HFHL and non HFHL) for HFHL_25_. Thirty-four workers had a HFHL for at least one ear, as determined by the reference test. In the first test, 24 of these workers with a HFHL were correctly identified by OEC (i.e., the true positives). In 17 workers, the OEC wrongly identified a hearing loss (i.e., the false positives). Twelve workers with HFHL were wrongly labeled as non-HFHL (i.e., the false negatives), while 29 non-HFHL correctly received a negative result (i.e., the true negatives). The sensitivity was 65%, and the specificity was 63%. When taking the results into account of the automatic conditional rescreen, sensitivity decreased to 59%, while specificity increased to 93%.

**Table 1:** Two-by-two contingency tables: HFHL_25_, for the single screening, and for the conditional rescreening

OEC result^a^	Pure-tone air conduction audiometry^b^		
	HFHL_25_	Non HFHL	Total
*Single screen*			
Positive	22	17	39
Negative	12	29	41
Total	34	46	80
*Conditional rescreen*			
Positive	20	3	23
Negative	14	43	57
Total	34	46	80

^a^Occupational Earcheck (OEC) result based on a cut-off value of − 14.9 dB SNR to discriminate between a positive result for at least one ear, and a negative result for both ears

^b^True high-frequency hearing loss for at least one ear (HFHL_25_) is defined as a pure-tone average of the frequencies 3, 4, 6 kHz (PTA_346_) of 25 dB HL or worse, according to the pure-tone air conduction audiometry test

Table 2 presents the OEC results compared to pure-tone air conduction audiometry results for HFHL_35_. Eighteen workers had a HFHL for at least one ear, as determined by the reference test. The sensitivity was 100% and the specificity was 66%. When taking the results into account of the automatic conditional rescreening, sensitivity decreased to 94%, and specificity increased to 90%.

**Table 2:** Two-by-two contingency tables: HFHL_35_, for the single screening, and for the conditional rescreening

OEC result^a^	Pure-tone air conduction audiometry^b^		
	HFHL_35_	Non-HFHL	Total
*Single screen*			
Positive	18	21	39
Negative	0	41	41
Total	18	62	80
*Conditional rescreen*			
Positive	17	6	23
Negative	1	56	57
Total	18	62	80

^a^Occupational Earcheck (OEC) result based on a cut-off value of − 14.9 dB SNR to discriminate between a positive result for at least one ear, and a negative result for both ears

^b^True high-frequency hearing loss for at least one ear (HFHL_35_) is defined as a pure-tone average of the frequencies 3, 4, 6 kHz (PTA_346_) of 35 dB HL or worse, according to the pure-tone air conduction audiometry test

Table 3 presents the association of the single screen versus the conditional rescreen, with the presence and absence of HFHL_25_ and HFHL_35_ for the total group, and the two age groups. For HFHL_25_ high positive likelihood ratios were found for the conditional rescreen in all workers (8.4), and for the age group > 45 years (8.1). For HFHL_35_ high positive likelihood ratios were found for the conditional rescreen in the total group (9.4), and for the younger age group (20). In addition, for HFHL_35_ low negative likelihood ratios were found in case of the conditional rescreen (0.07 for the total group, and 0.08 for the older group). High negative predictive values were particularly found for HFHL_35_, with and without the conditional rescreen.

**Table 3:** Association of single screen and conditional rescreen, and population (all, young, and old) with the presence and absence of HFHL_25_ and HFHL_35_, expressed as sensitivity, specificity, predictive values and likelihood ratios

Screen	Degree HFHL	Population	Sensitivity	Specificity	PV+	PV−	LR+	LR−
*Single screen*	HFHL_25_	All	65% (22/34)	63% (29/46)	56% (22/39)	71% (12/41)	1.8	0.6
		≤ 45 years	50% (4/8)	70% (23/33)	29% (4/14)	85% (23/27)	1.7	0.7
		> 45 years	69% (18/26)	46% (6/13)	72% (18/25)	43% (6/14)	1.3	0.7
	HFHL_35_	All	100% (18/18)	66% (41/62)	46% (18/39)	100% (41/41)	2.9	–
		≤ 45 years	100% (3/3)	71% (27/38)	21% (3/14)	100% (27/27)	3.4	–
		> 45 years	100% (15/15)	58% (14/24)	60% (15/25)	100% (14/14)	2.4	–
*Conditional rescreen*	HFHL_25_	All	59% (20/34)	93% (43/36)	87% (20/23)	75% (43/57)	8.4	0.4
		≤ 45 years	38% (3/8)	94% (31/33)	60% (3/5)	86% (31/36)	6.3	0.7
		> 45 years	65% (17/26)	92% (12/13)	94% (17/18)	57% (12/21)	8.1	0.4
	HFHL_35_	All	94% (17/18)	90% (56/62)	74% (17/23)	98% (56/57)	9.4	0.07
		≤ 45 years	100% (3/3)	95% (36/38)	60% (3/5)	100% (36/36)	20	–
		> 45 years	93% (14/15)	83% (20/24)	78% (14/18)	95% (20/21)	5.5	0.08

HFHL_25_ = high-frequency hearing loss for at least one ear, defined as a pure-tone average of the frequencies 3, 4, 6 kHz (PTA_346_) of 25 dB HL or worse. HFHL_35_ = high-frequency hearing loss for at least one ear, defined as a pure-tone average of the frequencies 3, 4, 6 kHz (PTA_346_) of 35 dB HL or worse

*PV+* positive predictive value, *PV−* negative predictive value, *LR+* positive likelihood ratio, *LR−* negative likelihood ratio

## Discussion

In this study, conventional pure-tone air conduction audiometry results were compared to results of the online speech-in-noise hearing screening test OEC for HFHL in a population of noise-exposed workers. For HFHL_25_, a moderate sensitivity of 65% and specificity of 63% was found. Automatic conditional rescreening significantly improved the specificity of the test to 93%. Especially, the older population seemed to benefit from a second chance, with an increase in specificity of 46–92%. Sequential testing seems to be beneficial as it further reduced the number of false positives. Although, testing duration increased, the total number of false positives incorrectly referred for further audiological assessment significantly decreased. The positive likelihood ratio of 8.4 indicates that OEC is particularly able to rule in HFHL_25_ with a reasonably high degree of confidence. In other words, if workers achieve a positive (i.e., poor) test score on OEC, it can be quite certain that they actually have HFHL_25_, as the majority of non-HFHL individuals would not have such high SRT results. On the other hand, the sequential rescreening lead to a deterioration in test sensitivity of 65–59%, especially in the younger population (50–38%), which indicates that part of the younger workers with a HFHL were still able to achieve a negative result on the rescreen. The lower negative predictive values indicate the uncertainty of the actual hearing status of the workers with a negative (i.e., good) score.

For the more moderate HFHL_35_, however, OEC is both highly sensitive and specific. The positive likelihood ratio of nearly 10 indicates that the OEC is able to rule in HFHL with a high confidence, while the negative likelihood ratio below 0.1 provides strong evidence that OEC is also able to rule out HFHL. Furthermore, with a positive likelihood ratio of 20, the OEC is strongly predictive of the detection of HFHL_35_ in younger workers.

Test accuracy was investigated for two age categories. The test sensitivity was lower in the younger population (except for HFHL_35_, after the conditional rescreen), while the specificity was lower in the older population. This implies that the younger workers were more often able to achieve a negative test result despite a HFHL, as compared to the older workers. This may be due to the severity of the HFHL, as the severity of the target condition determines the probability of finding positive test results (Moons et al. 1997). Age is associated with the severity of the HFHL; the older workers showed larger hearing losses as compared to the younger workers.

In an earlier evaluation of OEC in a noise-exposed population higher sensitivity and specificity values were found, even without rescreening, namely 90 and 77%, respectively (Sheikh Rashid et al. 2017b). This may be due to the fact that the cut-off point for pass/fail was derived post hoc from the same population, which may have overestimated the accuracy of the test. Furthermore, sensitivity and specificity values may vary across populations due to selection bias, as well as due to variations in population characteristics (Moons et al. 1997), including age, and the severity of the hearing loss. Leensen & Dreschler investigated the internet-based speech-in-noise test Earcheck, which is based on the same principles as OEC, and found a comparable moderate sensitivity of 68% and specificity of 71% in 249 male construction employees (mean age = 49.7 years) for one screening round (2013a). Jansen et al. compared the Digit Triplet test, a consonant–vowel–consonant test with words with the same vowel (CVC), and a CVC test with a low-pass filtered (CVC_LP) with high-frequency PTA in 118 noise-exposed workers (age range = 22–59 years) (2013, 2014). A higher sensitivity of 92%, and a specificity of 89% to detect mild HFHL (defined as a PTA_2346_ above 10 dB HL) was found for the Digit Triplet test (Jansen et al. 2013). For the CVC tests, an increased measurement error and a weaker correlation with PTA_2346_ was found as compared to the more reliable Digit Triplet test (CVC: *R* = 0.86, CVC_LP: *R* = 0.79, Digit Triplet: *R* = 0.86) (Jansen et al. 2014). These studies, however, did not account for different ages when investigating sensitivity and specificity. In addition, they did consider a single screening round only.

Sekhar et al. considered the effect of a two-step screening on test sensitivity and specificity in HFHL screening in adolescents (2016). State school-based hearing screens, threshold tests at 250–8000 Hz using pulsed pure tones conducted in the school library, were compared to the gold standard sound-treated booth testing. Initial referrals returned for repeated screening. Following the two test rounds, specificity improved (from 49.5% to 84.6%), while sensitivity maintained (76.7%). In the current study, specificity improved as well, however, sensitivity decreased slightly. In the study by Sekhar et al. (2016), the two test rounds of threshold testing only reduced the number of false positives, while for OEC, the number of false negatives increased as well. This may be well explained by the learning effect that OEC encounters.

An important limitation of this study was that the study participants were not randomly selected. The employees voluntarily participated in an occupational audiometric examination because they were more health conscious, or more worried about their hearing ability. This may have resulted into selection bias, affecting the prevalence and severity of HFHL. Therefore, the values of the test properties of OEC may differ in other noise-exposed populations. Another important limitation of this study is that one of the two audiometrists did not include the octave frequencies 3 and 6 kHz, which are important for the diagnosis of HFHL according to the audiogram. Therefore, the adjacent frequencies were interpolated for 60 of the 80 workers. As a consequence, the measurement accuracy of the high frequency test point 4 kHz weighted more heavily as compared to that of the other frequencies. Furthermore, it is not clear whether the HFHL in the workers was related to noise. It is important to note that the HFHL could have been a combination of noise-induced hearing loss and presbycusis. For the purpose of this study, the most important result is that OEC is able to discriminate between HFHL and non-HFHL, despite the actual cause of the hearing loss.

For further practice, it is important to consider the actual goal of screening with OEC in certain situations. According to this study, OEC appears to be quite suitable if the goal is to rule in/out moderate HFHL or worse, especially in younger populations. This means that OEC provides an important tool for the identification of individuals who are likely to benefit from preventive measures to prevent worsening of the hearing loss, or in more severe cases, from hearing aids. If the goal is, however, to screen for early/mild HFHL (HFHL_25_), OEC would probably miss out on a significant percentage of cases, but would be quite specific (i.e., low number of false positives). In that case, the chance that non-HFHL workers will have a positive result and unnecessarily be referred to further audiological assessment would be small. This may be cost efficient, as unnecessary expensive and invasive audiological diagnostic assessment can be avoided. The false negatives could possibly be detected in another screening round, for instance by means of annual screening. Future studies on OEC may, therefore, focus on (the potential learning effects on) periodic screening. Furthermore, future research may also focus more on variations in test accuracy parameters due to variations in (sub)populations, including differences in prevalence and severity of HFHL.

## Conclusions

In this study, the test accuracy of OEC for screening of HFHL in a noise-exposed population was validated. Automatic conditional rescreening seems to be beneficial, considerably improving test specificity. With a moderate test sensitivity of 59%, but a high test specificity of 93%, the test is particularly able to rule in mild HFHL_25_ with a reasonably high degree of confidence. OEC appears to be a more accurate screening test for higher degrees of HFHL (HFHL_35_), with a high test sensitivity of 94%, and a high test specificity of 90%. The accuracy of OEC may vary across different occupational noise-exposed populations. This should be explored further.

## Footnotes

1 The sensitivity of the test reflects the proportion correctly identified individuals with HFHL among all individuals with HFHL. The specificity reflects the proportion correctly identified non HFHL individuals among all non HFHL individuals.

2 The positive predictive value is the probability that the individual has hearing loss when OEC shows a positive result. The negative predictive value is the probability that an individual is non HFHL when OEC shows a negative result.

3 The positive likelihood ratio is the ratio of the probability of a positive OEC test in workers with HFHL to the probability in non HFHL workers. The negative likelihood ratio is the ratio of the probability of a negative OEC test in workers with HFHL to the probability in non HFHL workers.
